# Feasibility and Exploration of a Standardized Protocol for Cardiac CT Assessment of Rheumatic Mitral Disease

**DOI:** 10.31083/j.rcm2509322

**Published:** 2024-09-10

**Authors:** Zhou Liu, Yue Ren, Jiajun Liang, Yazhe Zhang, Hongkai Zhang, Maozhou Wang, Lei Xu, Yuyong Liu, Wenjian Jiang, Hongjia Zhang

**Affiliations:** ^1^Department of Cardiac Surgery, Beijing Anzhen Hospital, 100029 Beijing, China; ^2^Department of Radiology, Beijing Anzhen Hospital, 100029 Beijing, China; ^3^Beijing Institute of Heart, Lung and Blood Vessel Diseases, 100029 Beijing, China; ^4^Beijing Lab for Cardiovascular Precision Medicine, 100069 Beijing, China; ^5^Department of Cardiac Surgery, The First Affiliated Hospital of Anhui Medical University, 230022 Hefei, Anhui, China

**Keywords:** rheumatic mitral valve disease, cardiac CT evaluation, mitral valve repair, subvalvular apparatus, calcification assessment

## Abstract

Rheumatic mitral valve disease often requires surgical interventions, such as percutaneous mitral commissurotomy, surgical mitral valve repair, or replacement, especially in severe cases. This necessitates a precise preoperative assessment of the extent of mitral valve disease. Currently, transthoracic echocardiography, the gold standard for preoperative assessment, has limitations, such as restricted acoustic windows and dependence on the operator, which can affect the evaluation of subvalvular structures and calcification of the mitral valve. Previous studies have shown that cardiac computed tomography (CT), with its high resolution, strong multiplanar reconstruction capabilities, and sensitivity to calcifications, can effectively overcome these limitations. Therefore, this study aims to summarize and evaluate the effectiveness of cardiac CT in examining mitral valve leaflets, annulus, and subvalvular structures. It also reviews the feasibility and guiding significance of using cardiac CT to assess characteristic rheumatic mitral valve lesions.

## 1. Introduction

Rheumatic heart disease (RHD) is a complication of acute rheumatic fever caused by an abnormal immune response to Group A streptococci infection, leading to valve damage, particularly affecting the mitral valve. It affects over 40.5 million people globally, with an annual mortality of over 300,000 [1, 2]. Treatment options include percutaneous mitral commissurotomy (PMC) and surgical interventions like valve replacement or repair for severe cases [3]. Accurate preoperative assessment of valve structures and lesion severity is essential. Traditional echocardiography, while simple and convenient, has limitations including restricted imaging in the subvalvular region and reliance on examiner experience [4]. Due to advances in mitral valve surgery and interventional techniques, there is a growing need for accurate preoperative assessment of mitral valve structures. Cardiac computed tomography (CT), commonly used for assessing coronary artery disease, has shown significant benefits in visualizing valve structures and identifying lesions [5]. This article combines past clinical practices with research on using cardiac CT to evaluate rheumatic mitral valve disease, providing guidance for surgical treatments.

## 2. CT Evaluation of Normal Mitral Valve Anatomy

Traditional CT scans have high resolution for calcium deposits, while contrast agents improve visibility of mitral valve structures and blood flow. Electrocardiogram (ECG) gating reduces motion artifacts, and Cardiac CT with multiplanar reformation (MPR) technology helps locate abnormalities in the mitral valve for evaluation [6]. Comparative studies have shown that CT measurements of mitral valve structures are consistent with echocardiography and direct visualization during surgery [7, 8, 9].

### 2.1 The Method of Scanning and Collecting Images by Cardiac CT

After extensive practice, our center and imaging department have developed a specific scanning protocol for evaluating mitral valve disease using cardiac CT [10]. This includes retrospective ECG-gated scanning of the heart and collecting image data from the aortic arch to the diaphragmatic surface of the heart throughout the cardiac cycle. The scanning parameters include a tube voltage of 100kV and automatic optimization of tube current by the Smart mA. Rotation time: 0.28 s, pixel matrix: 512 × 512, collimation: 256 × 0.625 mm, the section thickness was 0.625 mm, with a reconstruction increment of 0.625 mm. scanning direction: head to foot. The effective radiation dose range was 5–15 mSv. Contrast injection: double-cylinder double-flow triphasic protocol. Lodopropamide contrast agent was injected at a rate of 4–5 mL/s for a total of 60–80 mL, with adjustments based on body mass index (BMI). The contrast was mixed with saline in a 3:7 ratio for a total of 30 mL in the second and third stages, with the same injection flow rate as the first stage. The contrast tracer method was used during the examination to select the region of interest (ROI) at the aortic root level. The scan started automatically after reaching a threshold of 100 HU (Hounsfield unit) in the ROI. The machine then automatically determined the best systolic and diastolic periods for image post-processing to obtain coronary artery information. Manual reconstruction by ECG editor when coronary artery observation is not ideal. Multiphase data reconstruction of the whole cardiac cycle at 10% intervals was used to observe aortic and mitral valve motion, with a reduced reconstruction range around the valves to minimize redundant data.

### 2.2 Mitral Valve Leaflets

**Fig. 1.**
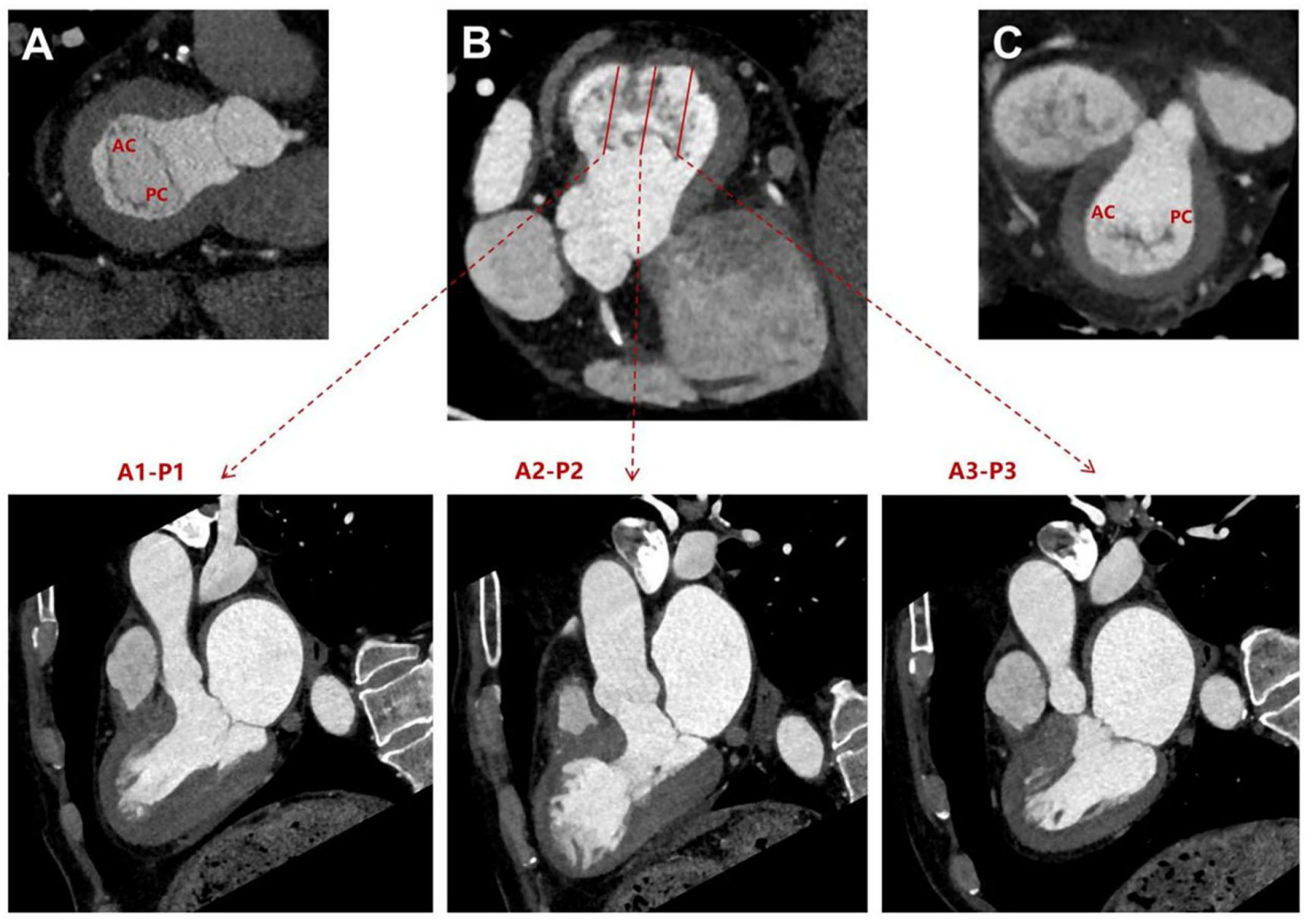
**Multiplanar reformation of the mitral valve leaflets after cardiac CT scan**. (A) Short-axis view of the mitral valve during diastole. (B) Long-axis views of the three regions of the mitral valve leaflets during systole. (C) Short-axis view of the mitral valve during systole. AC, anterior commissure; PC, posterior commissure; CT, computed tomography.

**Fig. 2.**
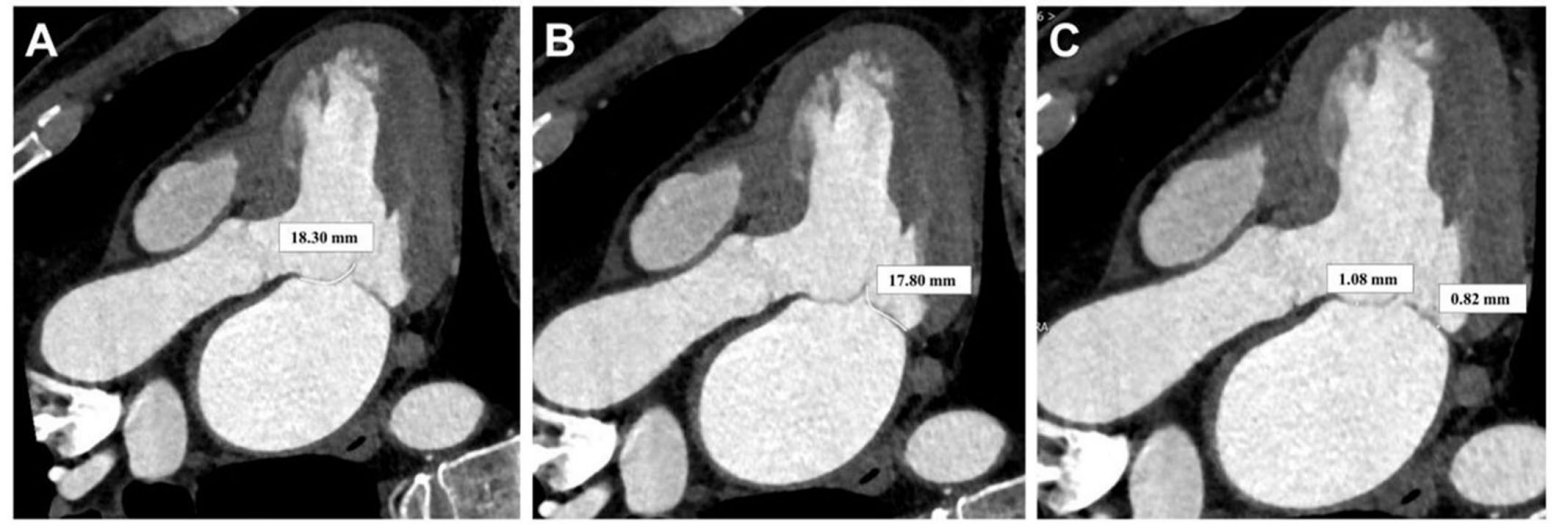
**Multiplanar reformation after cardiac CT scan for measuring the length and thickness of the mitral valve leaflets**. (A) Anterior leaflet length of the mitral valve. (B) Posterior leaflet length of the mitral valve. (C) Posterior leaflet length of the mitral valve. CT, computed tomography.

The mitral valve has anterior and posterior leaflets separated by commissures. The anterior leaflet is one-third of the circumference and more mobile, while the posterior leaflet is two-thirds of the circumference and less mobile [11]. The posterior leaflet is divided into segments P1, P2 and P3, and the anterior leaflet is divided into A1, A2 and A3. The boundary between A1 and P1 is known as the anterior commissure (AC), while the boundary between A3 and P3 is termed the posterior commissure (PC). Additionally, the anterior leaflet of the mitral valve, extending from the base to the free edge, is further divided into a thin, semi-transparent zone (the clear zone) and a thicker, non-smooth zone (the rough zone). The rough zone is positioned at the coaptation site of the leaflets during systole [12, 13]. As shown below, cardiac CT with MPR provides a clear depiction of the mitral leaflet regions and their junctions during systole or diastole (Figs. 1,2).

### 2.3 Mitral Valve Annulus

**Fig. 3.**
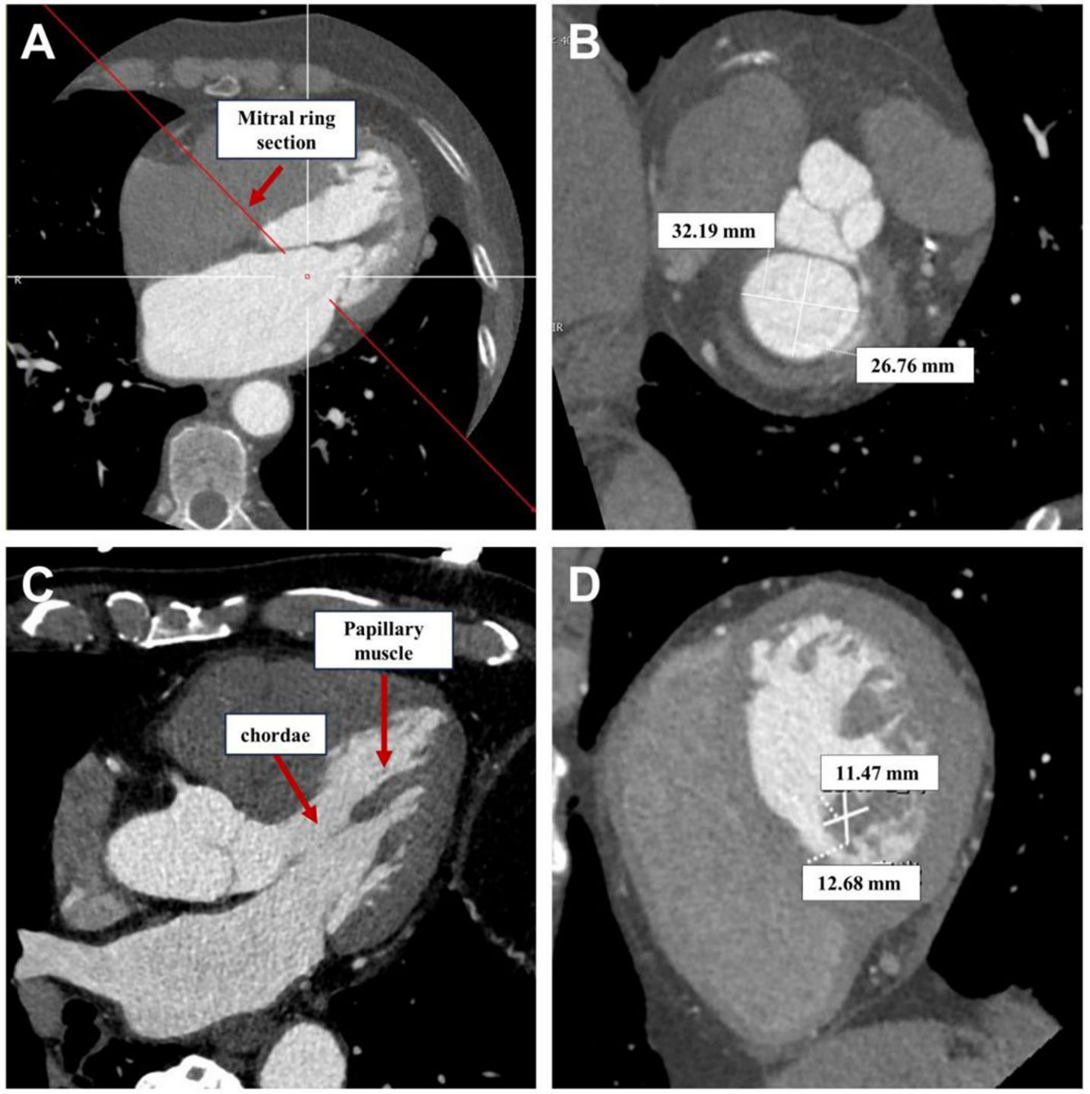
**Multiplanar reformation after cardiac CT scan for visualizing the mitral valve annulus, chordae tendineae, and papillary muscles**. (A) Long-axis view for positioning the mitral valve annulus. (B) Long-axis view for positioning the mitral valve annulus. (C) Long-axis view for positioning the mitral valve annulus. (D) Short-axis view for measuring the long and short diameters of the papillary muscles. CT, computed tomography.

The mitral valve annulus separates the left atrium and ventricle, supporting the leaflets with a saddle-shaped structure that changes shape during the cardiac cycle [14, 15]. The anterior annulus of the mitral valve is fibrous and less likely to dilate, while the posterior annulus is muscular and more prone to dilation and calcification in severe mitral regurgitation cases [12, 16]. As depicted in Fig. 3A,B, reconstructed images at the annular cross-section obtained from cardiac CT scans are instructive for visualizing and measuring the annular diameter (Fig. 3A,B).

### 2.4 Chordae Tendineae and Papillary Muscles

The subvalvular apparatus of the mitral valve includes the papillary muscles and chordae tendineae, forming the leaflet suspension system. Papillary muscles are divided into anterior and posterior groups, attached to the ventricular wall beneath the commissures [13]. Chordae tendineae connect papillary muscles to leaflets and are classified into basal, intermediate, and marginal types based on attachment locations [12, 13]. Our study shows that reconstructing subvalvular chordae and papillary muscles from cardiac CT scans is better than echocardiography (Fig. 3C,D). MPR of the ventricular long-axis and short-axis sections using CT provides clear visualization of subvalvular structures, facilitating measurements of papillary muscle and chordae lengths, as well as assessing the symmetry of papillary muscles.

## 3. Cardiac CT Evaluation of Rheumatic Mitral Valve Disease

Rheumatic valvular disease primarily affects the mitral valve, causing inflammation in the acute phase and leading to changes like leaflet thickening and calcification in the chronic phase, ultimately resulting in mitral stenosis [17]. As the disease advances, both stenosis and regurgitation can occur together [18]. Cardiac CT, with its clear MPR, is beneficial for visualizing and quantitatively evaluating the characteristic lesions of rheumatic mitral valve disease, offering a valuable assessment of the severity of stenosis and regurgitation.

### 3.1 Evaluation of Leaflets, Chordae Tendineae, and Papillary Muscles Lesions

**Fig. 4.**
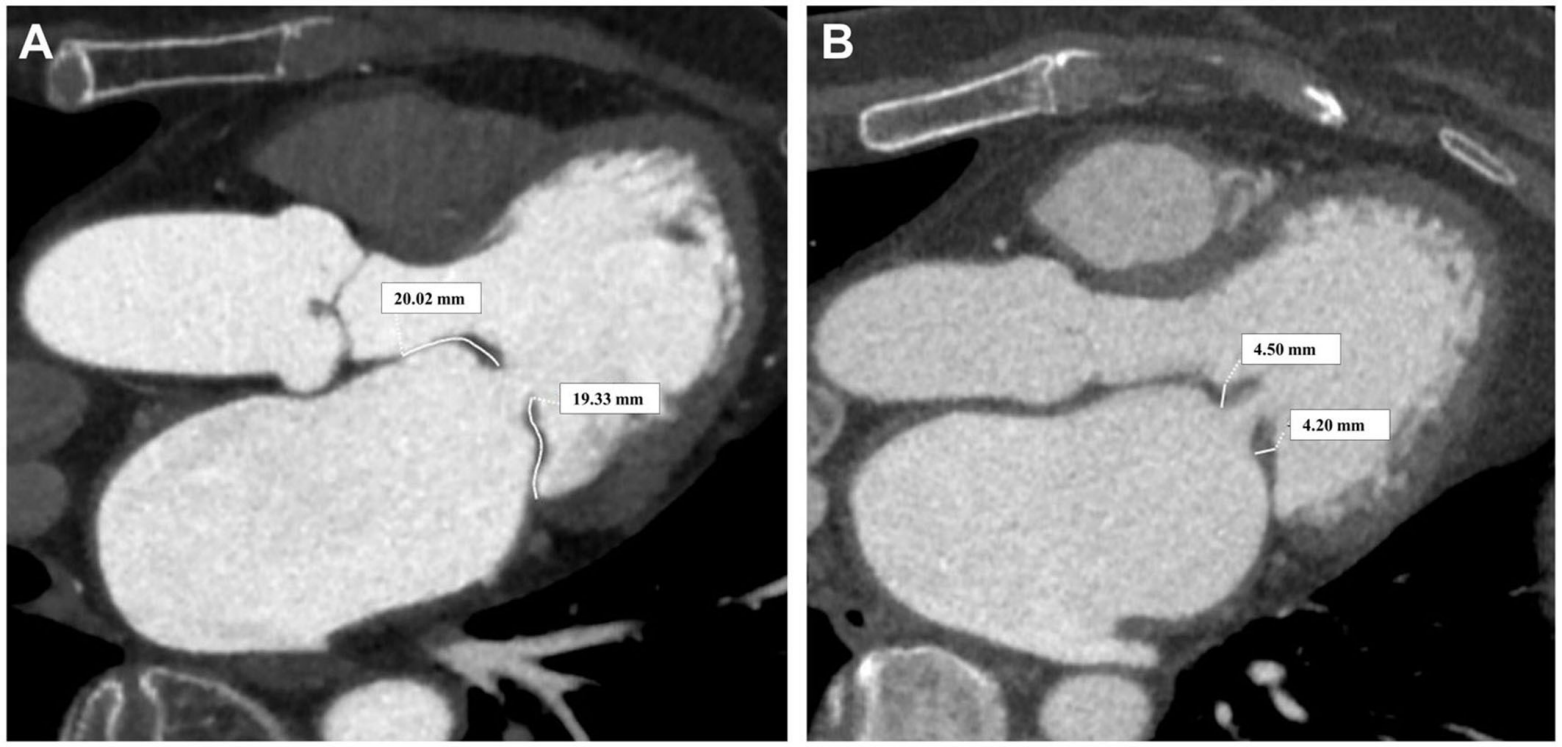
**Rheumatic mitral valve leaflets**. (A) Measurement of leaflet length. (B) Measurement of leaflet thickening degree.

**Fig. 5.**
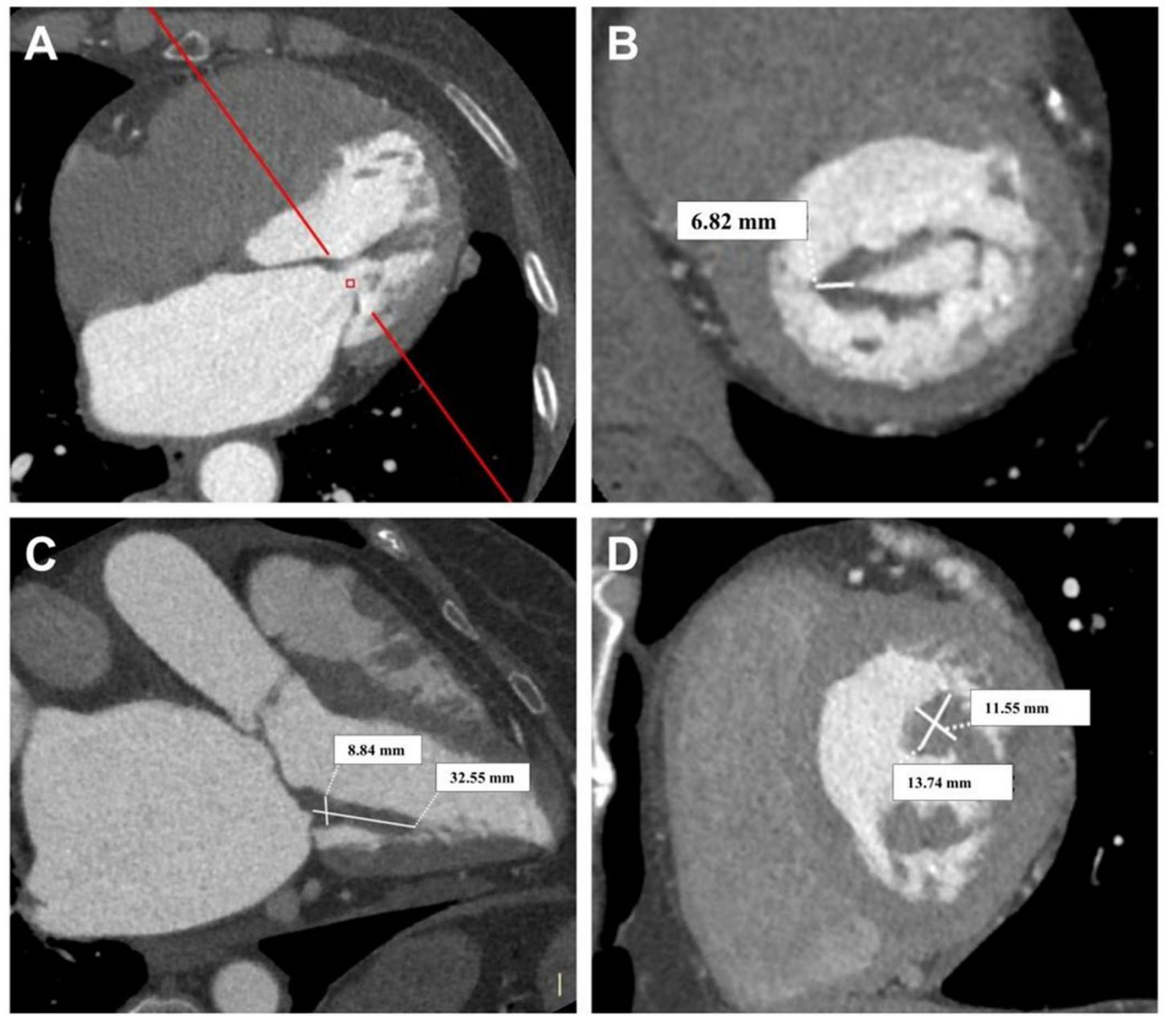
**Assessment of rheumatic mitral valve junction and subvalvular structures**. (A) Long-axis view of the valve orifice in the open position. (B) Short-axis view of the open valve orifice, assessing the degree of commissural fusion. (C) Long-axis view assessing the fusion of papillary muscle and chordae tendineae. (D) Short-axis view measuring papillary muscle length and diameter.

Severe rheumatic mitral valve disease in surgery candidates is characterized by thickened leaflets, fused commissures, and shortened chordae tendineae [13]. Cardiac CT can offer better visualization and measurement of these lesions, aiding in surgical planning. The assessment of leaflet thickening and contraction can be conducted on long-axis four-chamber views during diastole or systole, measuring the thickness and length of the three regions of the anterior and posterior leaflets. Fibrous thickening in the clear zone, which significantly impacts leaflet mobility, can be measured on the long-axis four-chamber view by selecting the 2/3 region near the base of the leaflet (Fig. 4). The measurement method is similar to echocardiography but with clearer boundary delineation. Assessing commissural fusion in rheumatic mitral valve lesions is crucial, as it impacts valve orifice area. CT scans can help measure adhesion length, indicating the degree of fusion (Fig. 5). Fusion and shortening of subvalvular structures can be evaluated during systole using long-axis and short-axis views, as shown in Fig. 5. The clarity of visualization and measurement accuracy are significantly superior to echocardiography [19]. Our center’s preliminary research suggests that CT measurement of the thickness of the thinnest part of the anterior leaflet’s clear zone (OR, 0.100; 95% CI, 0.023–0.439; *p* = 0.002) and the symmetry of the papillary muscles (OR, 0.964; 95% CI, 0.936–0.993; *p* = 0.016) significantly impact outcomes of early good mitral valve repair [10].

### 3.2 Assessment of Calcification

**Fig. 6.**
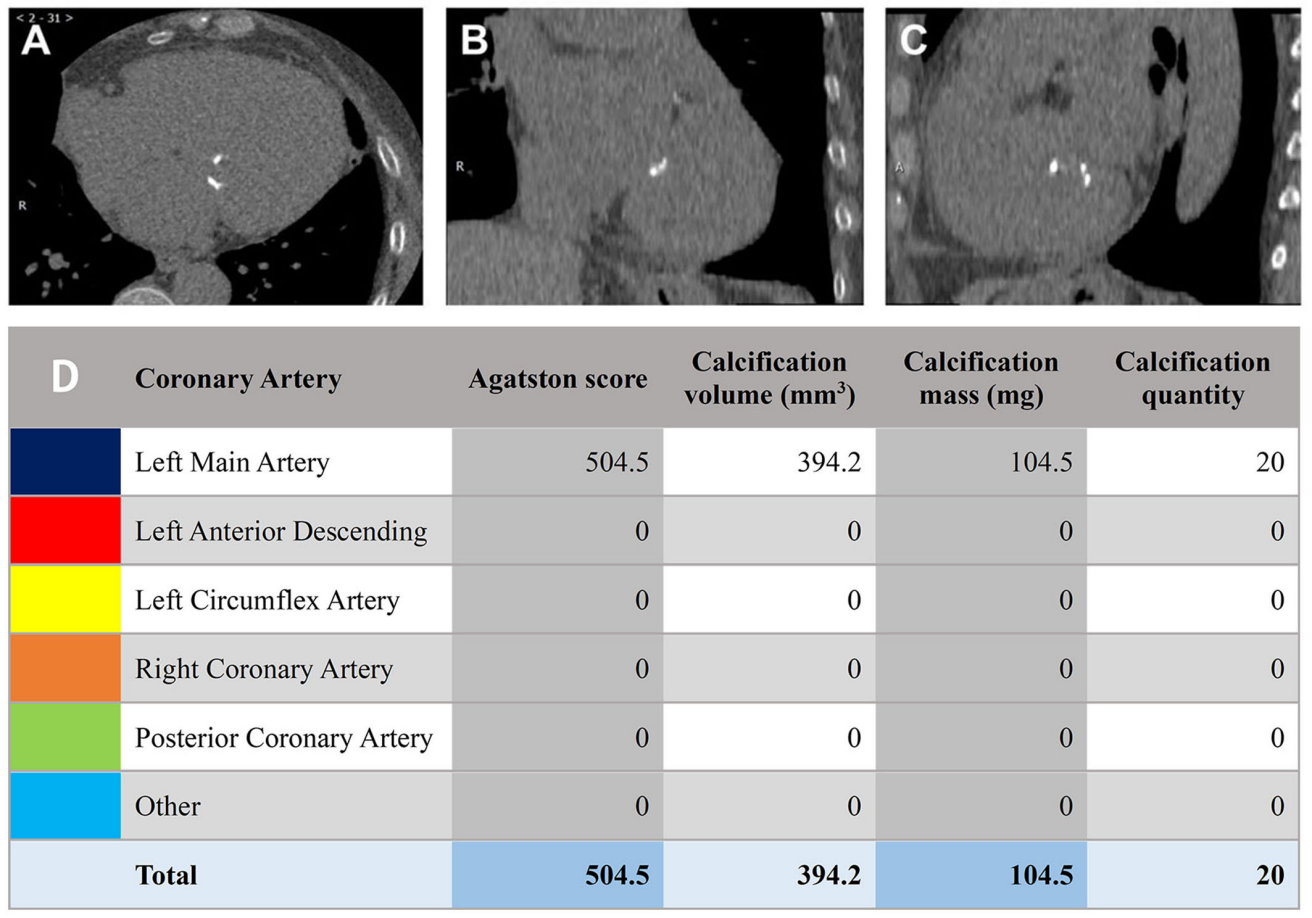
**Agatston scoring for quantifying mitral valve calcification**. (A–C) Frame-by-frame marking of calcification. (D) Agatston calculation of total calcification score, calcification volume, and calcification mass.

**Fig. 7.**
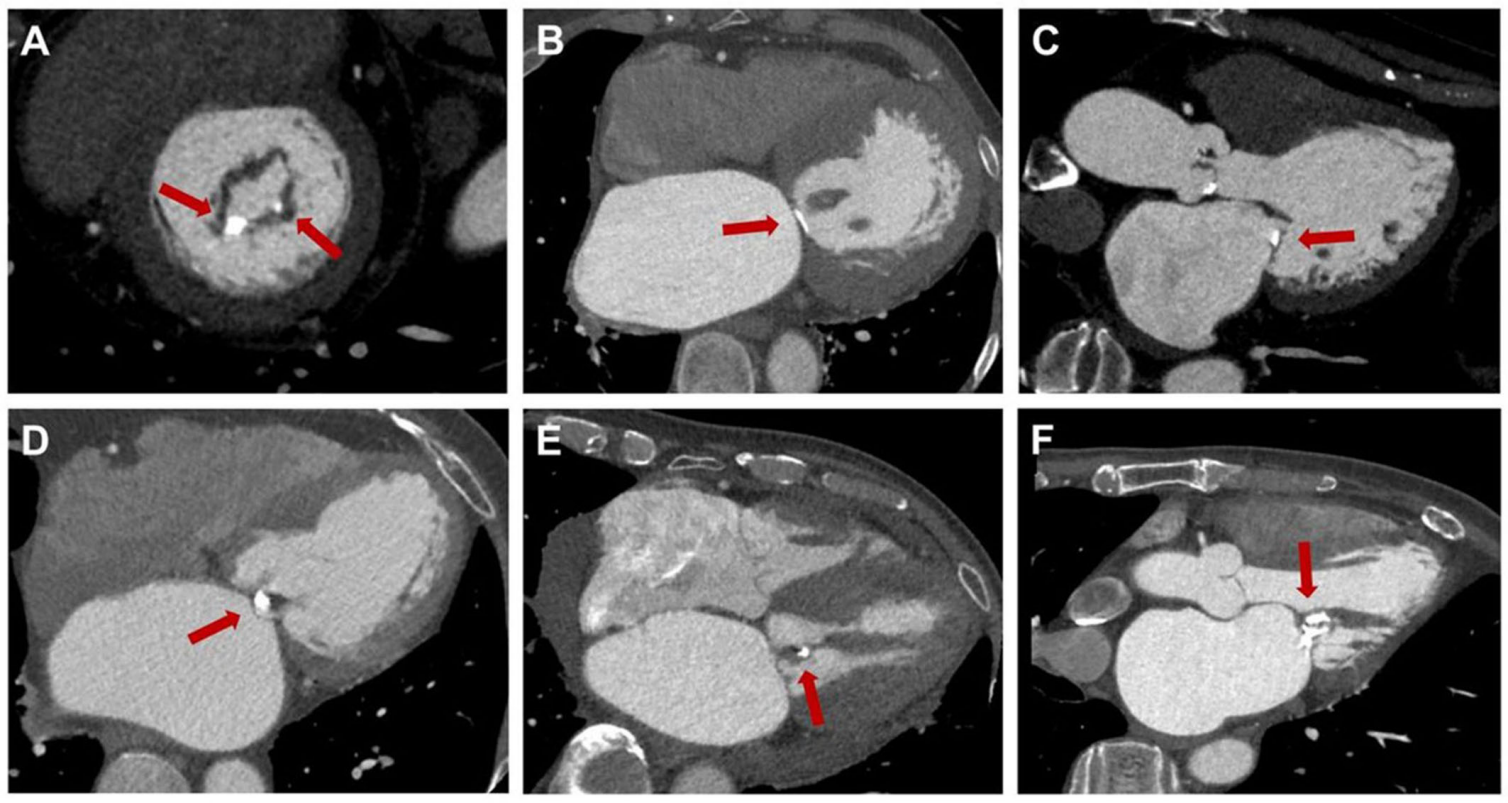
**Localization of rheumatic mitral valve calcification**. (A) Calcification at the posterior commissure, P2 region. (B) Calcification of the valve annulus. (C) Calcification of the posterior leaflet. (D) Penetrating calcification. (E) Chordae tendineae calcification. (F) Calcification involving valve leaflets, chordae tendineae, and papillary muscles. The red arrows highlight calcified plaques in various regions of the mitral valve.

In our early practice, we noticed a high rate of calcification in patients with rheumatic mitral valve disease, affecting about 59% of our surgical patients [10]. Unlike calcification in other types of mitral valve disease, calcification in rheumatic mitral valve disease is mainly found in the leaflets, commissures, and subvalvular structures [20, 21, 22]. This calcification has a significant impact on the prognosis of both PMC and surgical repair [22, 23, 24]. Accurate assessment of calcification is crucial for determining treatment strategies in patients with rheumatic mitral valve disease. Cardiac CT is superior to echocardiography in locating and quantifying calcification, especially when echocardiography results are unclear [25]. Mitral valve calcification found on CT can indicate mitral stenosis, guiding intervention strategies and leading to better patient outcomes [26]. The Agatston score, originally for coronary artery calcification, is also useful for quantitatively assessing mitral valve calcification severity on CT images (Fig. 6) [27]. Patients with higher Agatston scores may face more difficulties in mitral valve repair. CT evaluation of mitral valve annular calcification increases the likelihood of repair failure, replacement, and postoperative arrhythmias [20, 28]. MPR helps locate calcification and assess its impact on the mitral valve (Fig. 7). This includes evaluating leaflet calcification, commissural calcification, annular calcification, determining if calcification penetrates the leaflets, and assessing the extent of involvement of papillary muscles and chordae tendineae. In our previous study, a CT assessment system for the extent and location of calcification in rheumatic mitral valves was developed. Based on this system, we found that calcification quality scores and calcification in the anterior leaflet’s clear zone were significant independent risk factors affecting early successful repair in rheumatic mitral valve disease [10].

### 3.3 Assessment of Mitral Stenosis and Regurgitation

Mitral stenosis and regurgitation create abnormal hemodynamic states during the cardiac cycle, requiring dynamic assessment with echocardiography [29]. CT imaging can also be helpful in evaluating the severity of these conditions and guiding intervention timing [30]. CT-assisted orifice area measurements are better than MRI for accurately measuring mitral stenosis severity in patients with poor echocardiography imaging [31]. Post-mitral valve repair CT scans can show masses around the prosthetic ring that may cause functional mitral stenosis [32]. MPR can show the morphology of mitral regurgitation during systole, allowing for measurement of the regurgitant orifice area to determine severity [33]. CT assessment of isolated mitral regurgitation severity through ventricular volume measurements has shown good correlation with MRI and echocardiography estimates [34]. This method is not applicable when other valve diseases are present. CT measurements of mitral annulus diameter and evaluation of mitral annular calcification can predict improvement of mitral regurgitation after transcatheter aortic valve replacement (TAVR) [35]. The West China Hospital team simplified a D-shaped mitral annulus model using CT scans and found that the circumference of the D-shaped mitral annulus predicts improvement in mitral regurgitation after TAVR [36].

## 4. Cardiac CT Evaluation of Rheumatic Mitral Valve Disease

With the rapid development of intervention techniques, CT has become a standard examination for pre-assessment of TAVR [37], and its role in guiding intervention for mitral valve repair and replacement is evolving, primarily for degenerative conditions [38, 39, 40, 41]. However, there are few reports on the use of CT in the evaluation and guidance of rheumatic mitral valve disease. Patient selection for PMC in isolated mitral stenosis primarily relies on the Wilkins score and Cormier score based on transthoracic echocardiography [3, 42, 43]. Echocardiography limitations can lead to errors in evaluating rheumatic mitral valve disease, resulting in some unsuitable patients for PMC and a 15%–25% rate of adverse events and failure [44, 45, 46]. Therefore, for patients with poor acoustic windows or difficult-to-distinguish calcifications, preoperative CT reconstruction of the mitral valve structure as a supplement to echocardiographic evaluation is of significant importance. It helps to further screen out and exclude patients who are not suitable for PMC, avoiding adverse consequences [19, 47]. Preoperative CT reconstruction of the mitral valve structure is crucial for patients with poor acoustic windows or hard-to-see calcifications, as it can help identify unsuitable candidates for PMC and avoid adverse consequences.

Our center has extensive experience in surgically treating rheumatic mitral valve disease, with a standardized repair technique and classification system [48, 49, 50]. However, echocardiography may not always accurately guide surgical decisions, leading surgeons to rely on subjective visual assessment during procedures. CT scans can accurately assess mitral valve structures, helping to determine the severity of lesions before surgery. This information improves the success rate of mitral valve repair during surgery. CT measurements of leaflet thickness can guide leaflet thinning during surgery; sensitivity to calcifications can locate small calcifications preoperatively; three-dimensional (3D) reconstruction can guide removal of calcifications; and operations can restore flexibility and activity of mitral valve leaflets. CT can help surgeons identify calcifications that may increase the risk of leaflet perforation during surgery, impacting repair outcomes. It can also assist in selecting the appropriate prosthetic ring based on annular morphology and diameter [41]. A well-fitted prosthetic ring can improve repair outcomes by maximizing orifice area and maintaining proper leaflet movement and coaptation height [51, 52]. Preoperative measurements using CT can help predict post-repair results, especially in cases of leaflet contraction due to rheumatic lesions. Failure to maintain effective coaptation height after repair can lead to unsuccessful outcomes.

Accurate evaluation of subvalvular structures is essential for guiding the “loosening the subvalve” procedure in rheumatic mitral valve repair. Transesophageal echocardiography is not effective for visualizing subvalvular tendons and papillary muscles, but CT 3D reconstruction can provide a solution. Reconstructed images can accurately show tendon and papillary muscle fusion or contraction, as well as measure the length and fusion width of severely fused papillary muscles. This allows for assessment of the depth of cutting needed to restore normal tendon lengths during surgery. In a previous study, CT measurements of the long and short diameters of the anterior lateral and posterior medial papillary muscles were used to evaluate symmetry. Patients with uneven papillary muscles are at higher risk for residual regurgitation or repair failure after mitral valve repair. Due to the complexity of mitral valve subvalvular structures, subtle differences in the handling of papillary muscles or tendons can cause markedly different biomechanical effects, thereby affecting mitral valve motion, which is one of the difficulties that many surgeons face when attempting the “loosening the subvalve” step during rheumatic mitral valve repair [50, 53]. Preoperative CT measurements and evaluations can help surgeons perform more accurate and standardized operations. A predictive model based on CT evaluation and clinical factors was developed in a previous study to predict favorable early repair in rheumatic mitral valve disease. The model was validated externally and can help guide the selection of surgical strategies for these patients.

## 5. Conclusions

CT is a valuable supplement to transthoracic echocardiography for assessing mitral valve conditions due to its high resolution, large field of view, and quick results. Cardiac CT offers significant advantages in evaluating subvalvular structures and calcification in rheumatic mitral valve disease, as demonstrated in Table 1 (Ref. [4, 5, 54]). This study explores the feasibility of using cardiac CT to assess mitral valve structure and characteristics, introducing a standardized approach using contrast agents. This new strategy uses advanced cardiac CT data and cinematic rendering to improve preoperative exams and surgical planning for patients with rheumatic mitral valve disease. It will enhance evaluation protocols and outcomes, leading to a more precise and scientific approach to diagnosis and treatment of rheumatic mitral valve disease.

**Table 1.** **Comparison of cardiac CT and echocardiography in evaluating rheumatic mitral valve lesions [4, 5, 54]**.

	Echocardiography	Cardiac CT
Examination advantages	Simple, quick, dynamic imaging, good hemodynamics	High spatial resolution, stable imaging, strong multiplanar reconstruction capability, high sensitivity to calcification
Examination disadvantages	Limited acoustic windows, low resolution, significant subject influence	Contrast agents and radiation, poor hemodynamic assessment
Leaflet contraction (length measurement)	+++	+++
Leaflet thickening (thickness measurement)	+++	+++
Junction fusion (degree of fusion)	+++	+++
Leaflet mobility	+++	—
Chordal contraction (chordal length)	+	+++
Papillary muscle contraction (papillary muscle length)	+	+++
Calcification site	+	+++
Calcification degree (quantitative assessment)	—	+++
MS or MR degree	+++	+

“+” indicates weak assessment effectiveness, “+++” denotes excellent assessment effectiveness, and “—” signifies no assessment effectiveness. MS, mitral stenosis; MR, mitral regurgitation; CT, computed tomography.

## Abbreviations

CT, computed tomography; RHD, rheumatic heart disease; PMC, percutaneous mitral commissurotomy; MPR, multiplanar reformation; ROI, region of interest; AC, anterior commissure; PC, posterior commissure; TAVR, transcatheter aortic valve replacement; MS, mitral stenosis; MR, mitral regurgitation; ECG, electrocardiogram.
